# Developing a measure of distress-promoting parent behaviors during infant vaccination: Assessing reliability and validity

**DOI:** 10.1080/24740527.2018.1471325

**Published:** 2018-06-14

**Authors:** Rebecca Pillai Riddell, Hannah Gennis, Paula Tablon, Saul Greenberg, Hartley Garfield

**Affiliations:** a Psychology, Faculty of Health, York University, Toronto, Ontario, Canada; b Psychiatry Research, The Hospital for Sick Children, Toronto, Ontario, Canada; c Psychiatry, University of Toronto, Toronto, Ontario, Canada; d Pediatrics, Pediatric Medicine, University of Toronto, Toronto, Ontario, Canada; e Pediatric Medicine, The Hospital for Sick Children, Toronto, Ontario, Canada

**Keywords:** vaccination, pain, infant, caregiver, behavior

## Abstract

**Background:**

Infants rely on their parents’ sensitive and contingent soothing to support their regulation from pain-related distress. However, despite being of potentially equal or greater import, there has been little focus on how to measure distress-promoting parent behaviors.

**Aims:**

The goal of this article was to develop and validate a measure of distress-promoting parent behaviors for acute painful procedures (e.g., vaccinations) that could be used by researchers and clinicians.

**Methods:**

Following initial generation of measure items, focused group discussions were held with vaccinating clinicians to understand the measure’s face, content, and ecological validity. Archival video footage (*n* = 537 videos of infant-caregiver dyads during vaccination) was then coded using the measure of distress-promoting behaviors for 3 minutes post vaccine injection. Validity and reliability were examined using correlational analyses. Construct validity was assessed by convergent relationships with infant pain-related distress and divergent relationships were assessed with parent sensitivity and soothing-promoting behaviors.

**Results:**

The measure demonstrated both moderate to excellent interrater and test-retest reliability and convergent and divergent validity (absolute magnitude of *r*’s = 0.30 to 0.46).

**Conclusions:**

By demonstrating strong reliability and validity, this measure represents a promising new way to understand how caregivers interact with infants during painful procedures. Through focusing on distress promotion and using a format that may be coded both from video or in vivo, it is a feasible way to operationalize the impact of the caregiver on the infant’s pain experience in both research and clinical settings.

## RÉSUMÉ

**Contexte**: Les nourrissons dépendent de l’apaisement que peuvent éventuellement leur procurer leurs parents avec sensibilité afin de les soutenir dans la régulation de leur détresse liée à la douleur. Toutefois, malgré le fait qu’ils soient d’importance égale ou supérieure, peu d’attention a été accordée à la façon de mesurer les comportements parentaux qui favorisent la détresse.

**But**: Le but de cet article était de développer et de valider un outil de mesure de la détresse suscitée par les comportements parentaux dans le cadre de procédures entrainant une douleur aigue (ex.: la vaccination), qui pourrait être utilisée par les chercheurs et les cliniciens.

**Méthodes**: Suite à une première production d’outils de mesure, des groupes de discussion ont été menés avec des cliniciens faisant de la vaccination afin de comprendre la validité apparente, la validité de contenu et la validité écologique de l’outil de mesure. Des séquences vidéo d’archives (n = 537 vidéos de dyades nourrisson-prestataire de soins) ont ensuite été codifiées afin de mesurer les comportements qui favorisent la détresse au cours des trois minutes suivant l’injection du vaccin. La validité et la fiabilité ont été étudiées à l’aide d’analyses corrélationnelles. La validité de construit a été évaluée par les relations convergentes avec la détresse liée à la douleur du nourrisson, tandis que les relations divergentes ont été évaluées avec la sensibilité parentale et les comportements d’apaisement.

**Résultats**: L’outil de mesure a démontré un degré de fiabilité inter-évaluateurs et de fiabilité test-retest allant de modéré à excellent, ainsi qu’une validité convergente et divergente (valeur absolue de f 0,30 à 0,46).

**Conclusions:** En démontrant une grande fiabiilité et une grande validité, cet outil de mesure représente une nouvelle façon prometteuse de comprendre comment les prestataires de soins interagissent avec les nourissons pendant les procédures douloureuses. En mettant l’accent sur ce qui favorise la détresse et en ayant recours à un format qui peut être codifié à partir d’une vidéo ou in vivo, il est possible d’opérationnaliser l’effet du prestataire de soins sur l’expérience de douleur de l’enfant, que ce soit dans le cadre d’une étude ou dans un cadre clinique.

## Introduction

How a newborn infant learns to self-regulate from distress is highly dependent on caregiver behaviors during periods of distress. Although infants are born with some homeostatic self-regulatory capabilities, their caregivers’ contingent sensitive soothing can facilitate this process.^1–3^ Thus, measuring caregiver responsiveness and soothing behaviors has been an important component of infant acute pain studies for decades.^4–8^ One theory that helps us understand the infant-caregiver interaction during a painful event is attachment theory. Attachment theory purports that after the first year of life, reliable infant regulation patterns can be discerned based on watching how an infant responds to distress in the presence of his or her caregiver. These infant regulation patterns are strongly predicated on how the parent has behaviorally responded to the infant’s distress over the first year of life. A central premise of attachment theory is that parent behaviors that help a distressed infant are those that achieve closeness (proximity) and are contingent on the infant’s signaling.^9^ The behaviors that parents enact that exacerbate infant pain-related distress through limiting proximity or noncontingent responses are the subject of the current study.

Much of the literature on parenting in the pediatric pain context has focused on the effects of discrete soothing behaviors on young child pain responses.^4–8^ For example, research has shown that parental use of verbal reassurance (e.g., saying “It’s ok”) increases infant pain,^5,6^ whereas behaviors such as pacifying, rocking, and distraction have been shown to reduce pain-related distress.^4,6,7^ Of note, not all studies have found parent soothing behaviors to be effective in reducing infant distress. For example, one study found that maternal use of soothing behaviors such as holding, rocking, and stroking did not reduce behavioral and physiological distress following a painful procedure.^8^

Thus, when understanding parent behaviors in a painful context, it is not just about the quantity (i.e., the number of times a parent soothing behavior is enacted) but also the quality or sensitivity of parent behavior. Measures of the sensitivity of parent behaviors on infant distress have been shown to be consistently related to infant pain-related distress.^10–14^ However, parent sensitivity has only accounted for a moderate amount of the variance in infant pain-related distress, and parent sensitivity measures require extensive training, significant time commitments to code, and postgraduate knowledge in clinical and/or developmental psychology to achieve reliability. Moving in a new direction, the current study focuses on easily observable parent *distress-promoting* behaviors that could be coded during acute painful procedures, such as a vaccination appointment. Recent multivariate models suggest that when both coping-promoting and distress-promoting parental behaviors are concurrently examined in the same model, distress-promoting behaviors were more powerful determinants of pain-related distress in early childhood.^15^

### Present study

Using an attachment perspective, specific behaviors were generated that worked against proximity-seeking and contingent responding (i.e., behaviors that suggest ignoring, misunderstanding, or enhancing infant pain-related distress) to an infant’s distress. The purpose of this study was to develop and validate a feasible measure of distress-promoting behaviors that could be used for lab-based coding by researchers as well as in vivo coding by clinicians. A measure used within health settings should be appropriate to the *context* (e.g., vaccinating setting), must have *content* that is based on the current evidence-based practices and that is appropriate for all possible users (e.g., distress-promoting behaviors observed by researchers and clinicians), must demonstrate *usability* (i.e., cost effective, not overly time consuming), and must have an appropriate structure (i.e., logical and functional appearance).^16^ Using these priorities as our framework, we set out to answer two research questions:

1. What behaviors should be included in this measure based on both infant pain researchers and vaccinating clinicians’ experience and expertise?
2. What is the reliability and validity of this measure in the vaccination context?

We hypothesized that using an attachment lens to generate easily observable behaviors that worked against proximity and contingency would generate a list of distress-promoting behaviors that would have strong reliability and validity in an acute pain context (i.e., vaccination).

## Methods

### Procedure

Ethics approval was obtained from the affiliated university and tertiary pediatric hospital for the original OUCH cohort study. The method for obtaining the footage for coding are described extensively elsewhere.^11–13^ For the current study, there were two phases: item generation and preliminary psychometric evaluation.

#### Item generation

An initial set of parent behaviors (items) was anecdotally tracked during vaccination video coding from the OUCH cohort (>2300 vaccination episodes). Initial item generation discussions involved seven lab members who were reliable in the use of validated measures of caregiver emotional availability, discrete caregiver soothing behaviors, and/or infant pain-related distress. Once a subset of behaviors had been selected, the two lead authors (R.P.R. and H.G.) presented the items to vaccinating clinicians over three iterative discussion groups. In line with published recommendations on how to run these discussion groups,^17^ our discussion group size was manageable (less than 12 people), and open debate and discussion was encouraged. These three groups were held between July and September 2014, and each was between 60 and 75 minutes in duration. Table 1 outlines all changes that were made to the measure through these discussion group meetings, which included removal and merging of some original behaviors, enhancing behavioral descriptions, including a new behavior, as well as change to the structure of the measure for feasible use. Because saturation (i.e., no new ideas were being generated) had been achieved following the third meeting, no further meetings occurred, and the final eight behaviors were used for the second phase of the study.

**Table 1.** Item generation sequence with clinicians.

	Items	Key changes generated from clinicians	
Discussion group 1 (original meeting)	Caregiver behaviors: Frustration Fear/distress Flat face Fathom wrong Face cover Fashion first Flit away Fork over	Add behavior—*flee the scene*: Parent not present at any time during the needle Group caregiver behaviors into meaningful subcategories so they are easier to understand (i.e., face-related [frustrated, fearful face, flat face]; saying/doing to the infant [fathom wrong, face cover]; distancing from infant [flit away, fork over, flee the scene]) Clarify items using descriptions more understandable to professionals providing vaccinations Add pictures to make it easier to skim Clarify focus that infant had to be in high distress post-needle; 3 minutes post-needle	
Discussion group 2	Caregiver behaviors: Frustration Fear/distress Flat face Fathom wrong Face cover Fashion first Flit away Fork over Flee the scene	Create a separate sheet from the checklist with brief descriptions as a reminder Add one behavior—forceful—when you note that a parent is too rough with the child post-needle. Condensed all distance behaviors into one behavior to make it easier to score flit away	
Focus group 3 (final consensus)	Caregiver behaviors: Frustration Fear/distress Flat face Fathom wrong Face cover Fashion first Forceful Flit away	Consensus achieved on finalized format (checklist, cheat sheet) and content (behavioral definitions resonate with clinicians providing vaccinations)	

#### Psychometric evaluation

A total of 537 videos of 12-month vaccine injections were used to code the eight distress-promoting behaviors generated in phase 1. This study used data from the 12-month wave (*n* = 548) of the OUCH cohort.^12^ The 12-month wave was selected because the pattern of infant distress regulation based on parent behaviors is most reliable at this time.^9,18^ Due to video footage limitations in 11 dyads, a total of 537 dyads were included in the coding effort.

For reliability, 30% of the entire 12-month sample was coded by three separate raters (*n *= 161). In order to examine the stability of the presence of distress-promoting parental behaviors over time, a subsample of the parents from the 12-month vaccine injection was also coded for distress-promoting parent behaviors during their child’s 6-month vaccination appointment (*n* = 136). For validity, these videos had previously been coded for infant distress behaviors,^19,20^ caregiver emotional availability,^21^ and proximal soothing.^5^

### Measures

#### Parent demographic information

During the 12-month vaccination visit, caregivers completed a short demographic questionnaire inquiring about their relationship with the infant, education level, and self-reported heritage culture.

#### Parent distress-promoting behaviors

The measure developed from this study included eight distress-promoting behaviors. To help create a coding mnemonic for the behaviors, all eight behaviors in the final set began with the same letter. The behaviors included fathom wrong (i.e., making comments toward the highly distressed infant that do not address or discredit the infant’s distress, such as “It’s not so bad”), face cover (i.e., covering a screaming infant’s face with any object such as a hand or blanket), fashion first (i.e., dressing a highly distressed infant with no attempt made to soothe infant), forceful (i.e., handling the infant roughly, such as pulling a supine infant across the examining table with their wrist), frustration (i.e., any facial expressions that reflect irritation with the infant’s distress, such as rolling eyes, sighing), fearful (i.e., any parental facial expression that suggests that they are scared or frightened), flit away (i.e., any behavior or parental positioning that does not bring the infant close to the parent when the infant is in moderate to high distress), and flat face (i.e., complete lack of emotional expression in response to infant’s moderate to high distress), a rare but established distressing behavior for infants.^22^

With the exception of forceful, which is coded if seen at any time during the vaccination appointment, all of these parent behaviors were only coded if the parent engaged in these behaviors while the child was in moderate to high distress. Moderate to high distress was determined based on the presence of a full-lunged cry.^19^ The exception to this rule is forceful because the strong use of force by a parent, whether the infant is in distress or not, would promote distress. Scores ranged from 0 to 8, with higher scores indicating a greater number of distress-promoting behaviors. Behaviors were coded for 3 minutes after the last needle. To facilitate in vivo coding, the presence or absence of each behavior was used, not frequency counts of how many times each behavior was coded. Reliability and validity on the set of behaviors are presented in the Results section.

#### Infant pain-related distress

Two different behavioral pain measures were analyzed to determine convergent validity with the distress-promoting behaviors. Higher scores on both measures reflect higher pain-related distress. Both measures provided an assessment of the infant’s initial reactivity and regulation (i.e., return to homeostasis) from the needle, given the distinct differences between how an infant first responds to a stimulus (more reflexive) and how a child regulates from a painful stimulus (more deliberate).^23^ In order to substantiate the distress-promoting behaviors that promote pain-related distress, there would need to be a relationship such that a greater total of distress-promoting behaviors would be related to higher pain scores. The Modified Behavior Pain Scale (MBPS)^19^ is a measure of broad distress behaviors and includes the sum of three behavioral scales—Facial Expression (0–3), Cry (0–4), and Body Movement (0–3)—to depict the degree of infant pain-related distress on a scale of 0–10. Higher scores indicate greater pain. For study purposes, we looked at MBPS scores from four different time points: for the initial 15 seconds post-needle (MBPS needle), for 15 seconds one minute after an initial 15-second epoch (MBPS 1 min), for 15 seconds 2 minutes after the initial 15-second epoch (MBPS 2 min), and for 15 seconds 3 minutes after the initial 15-second epoch (MBPS 3 min). The MBPS has demonstrated strong concurrent and construct validity, as well as item total and interrater reliability within the immunization context.^19,24,25^ In the present study, interrater reliability ranged from 0.93 to 0.96.

The Neonatal Facial Coding System (NFCS)^20^ is a measure based on the specific facial constellation to painful stimuli, demonstrating content, construct, convergent, and face validity.^26^ It uses brow bulge, eye squeeze, nasolabial furrow, open lips, vertical stretch mouth, horizontal stretch mouth, and taut tongue to create a facial pain score. Each facial action is coded as 0 (*not present*) or 1 (*present*).^27^ Pain scores were obtained by calculating the proportion of time the facial actions were present for every second in a 10-second epoch following the needle (NFCS needle), for 10 seconds 1 minute after last needle (NFCS 1 min), for 10 seconds 2 minutes after last needle (NFCS 2 min), and for 10 seconds 3 minutes after last needle (NFCS 3 min). Scores range from 0 to 1, with higher scores indicating greater facial pain expression. In the present study, interrater reliability ranged from 0.85 to 0.97 for each facial action.

#### Caregiver emotional availability and proximal soothing

To examine divergent validity, two well-validated measures of caregiver soothing-promoting behavior were coded, one relating to the quality of caregiving and the other related to the quantity of close contact or proximal soothing behaviors (i.e., rocking and physical comforting) that facilitate secure attachment. Thus, we set out to demonstrate that the more distress-promoting behaviors the parents enacted, the lower the sensitivity and the amount of proximal soothing.

The Emotional Availability Scales–4th Edition (EAS)^21^ is a global measure of the quality of caregiving behaviors that has demonstrated construct and criterion validity.^28^ It examines caregiver behaviors on four different subscales: Sensitivity, Structuring, Non-intrusiveness, and Non-hostility. Through subscales that take into account the infant’s responses to the parent’s behavior, a clinical rating is made. High scores reflect greater caregiver sensitivity. Caregivers received a total score by combining the four subscales (range = 28–116). In the present study, interrater reliability for the total EAS scores ranged from 0.88 to 0.93.

The Measure of Adult and Infant Soothing and Distress (MAISD)^5^ has shown reliability and concurrent validity as an observation scale developed to evaluate behaviors of children, parents, and nurses during painful medical procedures. For the purposes of the present study, relationships with behaviors that could be considered proximal soothing were analyzed: rocking and physical comforting. Rocking and physical comfort were coded as present (1) or absent (0) in 5-second epochs for the 1-minute period after the last needle (MAISD rock 1 min, MAISD phys comf 1 min), for the 2-minute period after the last needle (MAISD rock 2 min, MAISD phys comf 2 min), and for the 3-minute period after the last needle (MAISD rock 3 min, MAISD phys comf 3 min). Index scores were calculated based on the proportion of time each behavior was present out of the total number of epochs that were codeable in a time period. Index scores ranged from 0 to 1, with higher scores indicating greater frequency of that behavior. Reliability coefficients across coders was strong to excellent, ranging from 0.91 to 0.95 for rocking and 0.75 to 0.88 for physical comfort.

### Analysis plan

To determine whether the measure was reliable, interrater reliability was measured using interclass correlation. In order to examine reliability over time, subsamples of distress-promoting parental behaviors from the 6-month and 12-month vaccination appointments were also compared.

To determine the measure’s construct validity, Pearson correlation coefficients were used to assess the convergent relationships between the total number of distress-promoting parent behaviors and the MBPS and NFCS scores immediately following the vaccine injection and at 1, 2, and 3 minutes post vaccine injection. Divergent relationships were also assessed using Pearson correlation coefficients between total number of distress-promoting parent behaviors and the EAS score and MAISD (rocking and physical comfort) scores 1, 2, and 3 minutes post vaccine injection. Due to a high number of correlations run, a Bonferroni correction was used (familywise error = 0.10; 0.10/14 correlational analyses = 0.007). Table 2 reports the interrelationships between all of the study variables.

**Table 2.** Correlations of total insensitive behaviors with measures of caregiver behaviors and infant distress.

	Total insensitive behaviors	MBPS needle	MBPS 1 min	MBPS 2 min	MBPS 3 min	NFCS needle	NFCS 1 min	NFCS 2 min	NFCS 3 min	EAS	MAISD rock 1 min	MAISD rock 2 min	MAISD rock 3 min	MAISD phys comf 1 min	MAISD phys comf 2 min	MAISD phys comf 3 min
Total behaviors	—															
MBPS needle	0.35***	—														
MBPS 1 min	0.42***	0.38***	—													
MBPS 2 min	0.46***	0.33***	0.52***	—												
MBPS 3 min	0.33***	0.28***	0.37***	0.51***	—											
NFCS needle	0.31***	0.53***	0.31***	0.24***	0.16**	—										
NFCS 1 min	0.36***	0.24***	0.51***	0.38***	0.25***	0.33***	—									
NFCS 2 min	0.30***	0.22***	0.28***	0.42***	0.26***	0.16**	0.35***	—								
NFCS 3 min	0.20***	0.14**	0.26***	0.36***	0.51***	0.15**	0.29***	0.23***	—							
EAS	−0.40***	−0.20***	−0.20***	−0.22***	−0.21***	−0.12**	−0.20***	−0.18***	−0.15**	—						
MAISD rock 1 min	0.02	0.13**	0.17***	0.19***	0.14**	0.12**	0.09*	0.11*	0.08	0.09	—					
MAISD rock 2 min	−0.05	0.12**	0.18***	0.14**	0.17***	0.16***	0.09*	0.09*	0.13**	0.01	0.55***	—				
MAISD rock 3 min	−0.05	0.15**	0.14**	0.09	0.14**	0.09	0.10*	0.10*	0.05	−0.02	0.36***	0.61***	—			
MAISD phys comf 1 min	0.02	0.10*	0.14**	0.05	0.01	0.18***	0.03	0.07	0.02	0.14**	0.36***	0.24***	0.09	—		
MAISD phys comf 2 min	0.04	0.11*	0.19***	0.14**	0.14**	0.13**	0.14**	0.14**	0.09	0.04	0.27***	0.41***	0.27***	0.43***	—	
MAISD phys comf 3 min	−0.01	0.03	0.11*	0.09	0.09	0.12**	0.11*	0.11*	0.09	0.05	0.18***	0.28***	0.36***	0.29***	0.46***	—

The correlations of interest in this table are those assessing the relationships between the total insensitive behaviors and measures of caregiver soothing behaviors (MAISD), emotional availability (EAS), and infant pain-related distress (MBPS, NFCS).

*P < 0.05. **P < 0.01. ***P < 0.001

MBPS = Modified Behavior Pain Scale; NFCS = Neonatal Facial Coding System; MAISD = Measure of Adult and Infant Soothing and Distress; EAS = Emotional Availability Scales.

## Results

### Demographic data

The average age of caregivers coded for this study was 34.09 years (SD = 5.16), and 86.9% of caregivers were mothers. They self-reported a diverse array of cultural backgrounds (37.6% European, 16.1% Asian, 12.1% North American, 7.6% Jewish, 6.5% Middle Eastern/African, 3.2% Latin/South American, 8.2% other, and 8.7% mixed), and most reported having an undergraduate degree or more (73.8% university degree or higher).

### Item development and face, ecological, and content validity

Clinicians and researchers came to consensus about the final items for inclusion (see Table 3). The participants agreed that the final content of the measure reflected distress-promoting behaviors (face and content validity) that are commonly seen during routine vaccination across their practices (ecological validity) and believed that the final measure was useable, with a clear structure and images that could be used in both research and clinical settings.

**Table 3.** Descriptions of behaviors.

Caregiver behavior	Description
Frustration	Parent expressed any sign of frustration at the infant’s high/moderate distress (e.g., sighing, eye rolling), or verbally expresses frustration (e.g., “Oh come on, just calm down”)
Fear/distress	Parent’s face looks scared or nervous around the needle/doctor or verbally expresses fear (e.g., “Oh, I hate needles—they are awful,” “needles are scary”) when infant is in high/moderate distress
Flat face	Parent shows no emotion (positive or negative) throughout the vaccination and particularly in response to infant’s high/moderate distress
Fathom wrong	When the infant is in high/moderate distress, the parent makes a statement that does not reflect the infant’s high distress level (e.g., saying, “You’re fine” over three times to a screaming infant, laughing at infant who is turning red from crying)
Face cover	When the infant is in high/moderate distress, the parent tries to cover his or her face (or mouth or eyes) with her hand, a blanket, etc.
Fashion first	When the infant is in high/moderate distress, the parent begins to dress the infant
Forceful	At any time, the parent uses excessive force with the infant (e.g., lifts the infant by the arms, puts infant down in a rough manner, pulls a supine infant across the table by the wrist)
Flit away	When the infant is in high/moderate distress, parent (1) puts the infant down, (2) holds infant away from her, (3) passes the infant off to someone else, or (4) is purposefully outside the room while child has the vaccination

### Descriptive statistics

The mean caregiver total of distress-promoting behaviors was 1.47 (SD = 1.10). The observed scores ranged from 0 to 5 (total possible score of 8). Of the entire sample, 22.5% had a score of 0, 30.7% had a score of 1, 26.3% had a score of 2, 18.2% had a score of 3, 2% had a score of 4, and 0.2% had a score of 5. Of the eight behaviors, the most commonly coded behavior was fathom wrong (53.8%), and the least common behavior was flat face (occurring in only 1% of the sample). Table 4 shows descriptive statistics for all other study variables.

**Table 4.** Means and standard deviations for infant pain-related distress, caregiver sensitivity, and sensitive soothing behaviors.

	Mean (SD)	Possible scale range
NFCS needle	0.73 (0.22)	0–1
NFCS 1 min post-needle	0.33 (0.24)	0–1
NFCS 2 min post-needle	0.26 (0.27)	0–1
NFCS 3 min post-needle	0.21 (0.18)	0–1
MBPS needle	8.26 (1.15)	0–10
MBPS 1 min post-needle	5.59 (2.49)	0–10
MBPS 2 min post-needle	4.79 (2.57)	0–10
MBPS 3 min post-needle	4.17 (2.50)	0–10
EAS	92.83 (10.29)	28–116
MAISD rocking 1 min post-needle	0.37 (0.32)	0–1
MAISD rocking 2 min post-needle	0.20 (0.29)	0–1
MAISD rocking 3 min post-needle	0.12 (0.24)	0–1
MAISD physical comfort 1 min post-needle	0.31 (0.26)	0–1
MAISD physical comfort 2 min post-needle	0.16 (0.21)	0–1
MAISD physical comfort 3 min post-needle	0.11 (0.20)	0–1

MBPS = Modified Behavior Pain Scale; NFCS = Neonatal Facial Coding System; EAS = Emotional Availability Scales; MAISD = Measure of Adult and Infant Soothing and Distress.

### Reliability

Interrater reliability between three independent coders for this study was excellent (average intraclass correclation coefficient = 0.92, *P* < 0.001; coder 1 with coder 2 = 0.93, *P* < 0.001, coder 1 with coder 3 = 0.89, *P* < 0.001). Further, distress-promoting parent behaviors were coded on a subsample of the same parents during the 6-month (*n *= 136) vaccine injection to examine the stability of distress-promoting behaviors over time. The relationship showed a medium effect size (*r* = 0.36, *P* < 0.001; *d* = 0.77).

### Construct validity (convergent and divergent validity)

#### Infant pain scores

The total of distress-promoting behaviors was strongly correlated with the MBPS and NFCS immediately following and in the minutes post vaccine injection. Moderate to strong positive relationships were seen between the total number of distress-promoting behaviors and the MBPS immediately following the needle (*r *= 0.35, *P* < 0.001; medium effect size *d* = 0.75), 1 minute post vaccine injection (*r* = 0.42, *P* < 0.001; large effect size *d* = 0.93), 2 minutes post vaccine injection (*r* = 0.46, *P* < 0.001; large effect size *d *= 1.04), and 3 minutes post vaccine injection (*r* = 0.33, *P *< 0.001; medium effect size *d* = 0.70). Strong positive relationships were also seen between the total distress-promoting behaviors and NFCS immediately following the needle (*r *= 0.31, *P *< 0.001; medium effect size *d = *0.65), 1 minute post vaccine injection (*r* = 0.36, *P* < 0.001; medium effect size *d *= 0.77), and 2 minutes post vaccine injection (*r *= 0.30, *P *< 0.001; medium effect size *d* = 0.63).

#### Caregiver behavior scores

A strong negative relationship was seen between the total distress-promoting behaviors and EAS (*r *= -0.40, *p* < 0.001; large effect size *d* = 0.87). Significant relationships were not seen between the total distress-promoting behaviors and the MAISD caregiver proximal soothing and rocking subscales at any time point. Table 2 displays all convergent and divergent relationships.

## Discussion

Infants heavily rely on their parents’ sensitive responses to regulate their pain-related distress.^1–3^ According to attachment theory, distressed infants signal to their parents to bring them close and elicit caregiving.^9^ To soothe their infant’s distress sensitively, parents need to be attuned to their signaling and maintain close proximity, with ongoing monitoring of the infant’s changing needs, alongside flexible responding to these changing needs. Though parent soothing and caregiver sensitivity has been extensively examined in the pediatric pain literature and has been shown to reduce infant pain-related distress,^11-14^ a large amount of variance in infant pain behaviors is still left unaccounted for. Given that no tools exist that operationalize parent behaviors that promote pain-related distress in infants, the goal of this study was to develop and validate such a measure.

### Interpretation of findings

This measure demonstrated moderate to strong interrater and test-retest reliability. There was high agreement on the total number of distress-promoting behaviors present between coders, and there was a moderate relationship between the total of these parent behaviors at the 6- and 12-month vaccination appointments. It is important that two of the three coders were undergraduate students who had less than 1 year of pain-specific research experience when learning the measure, because this suggests the ease at which these behaviors can be learned.

The final structure of this measure included eight distress-promoting behaviors. This was based on in-depth discussions between researchers with experience coding parent behaviors during vaccination, as well as health care professionals responsible for vaccinations. Through three focused discussion groups with clinicians, we were able to create an ecologically valid measure with content and face validity. Involving both researchers and clinicians who provide vaccinations in the development phase was critical because we strove to have feasibility in both research and clinical settings.

As hypothesized, our measure was shown to be a reliable and valid way to measure parent distress-promoting behaviors using archival vaccination footage. Construct validity was shown through convergent relationships with infant pain measures. Moderate to strong convergent relationships were found between the number of distress-promoting behaviors and the two separate measures of infant pain-related distress post vaccine injection. The more distress-promoting behaviors the parents enacted, the greater the infant’s pain-related distress. These relationships were strongest following the needle, 1 minute following the needle, and 2 minutes following the needle and confirm the importance of coding these distress-promoting behaviors when the infant is in moderate to high distress. In addition, there was a strong divergent relationship between the total number of distress-promoting behaviors and caregiver emotional availability, suggesting that the more distress-promoting behaviors present, the less emotionally available or sensitive the caregiver was in the minutes following the needle puncture.

Interestingly, no relationships were found between distress-promoting parent behaviors and discrete soothing parent behaviors. This could be due to the previously discussed issue that the higher frequency of a soothing behavior (i.e., how many times rocking and physical comforting occurred) may not always be what a particular infant wants in that moment (i.e., contingency). Thus, high scores and low scores on the soothing measure could mean exactly the same thing for different infants within the sample. This was not the case for our set of distress-promoting behaviors. The behaviors were carefully selected because they were consistently distress-promoting in our sample when done to an infant in moderate to high distress. Thus, the higher the number of distress-promoting behaviors, the greater the presence of a variety of distress promotion responses (i.e., coders did not count how many times a specific distress-promoting behavior occurred, just that it occurred). The very strong inverse relationship found with a clinical judgment of caregiver sensitivity (i.e., the EAS, the measure that takes into account the impact of those behaviors on the infant and thus higher scores always mean higher sensitivity) adds strength to this speculation.

### Implications and future directions

To our knowledge, this is the first measure to focus specifically on distress-promoting behaviors. One of the primary benefits of creating and validating this measure is finding a new way to assess the impact of the parent on his or her infant’s pain responding. Parent soothing and sensitivity have been studied in the pediatric pain literature, measuring that these constructs present with their unique challenges. First, frequency counts of soothing behaviors lack a demonstration of whether particular soothing behaviors are attuned to the infants’ needs (i.e., sensitivity). Second, measures of parent sensitivity are time consuming to learn, often need to be learned from the original developers of the scale due to the nuance of its coding (it is generally seen as a clinical judgment measure), are costly to maintain reliability in a lab over time, and often require a high level of health professional or clinical psychology graduate training to become successfully reliable. The current measure of easily observable distress-promoting behaviors combines the feasibility of coding the presence of a behavior in vivo, with an emphasis on behaviors known not to be attuned to the infant’s needs.

This measure therefore can benefit a wider range of scientists, as well as clinicians. In terms of research use, providing nonclinical scientists with a feasible measure of parenting behaviors allows for a more thorough exploration of their research questions (e.g., the confounding parent variable on treatment effects). Further, behavioral scientists would benefit from a new way to measure parent behavior, with the possibility of accounting for more variance in infant pain responding. Finally, there is great potential for incorporation into primary care by clinicians. By teaching health professionals responsible for vaccinations to look for these distress-promoting behaviors, clinicians will be better able to coach parents in the immediate moment on different strategies that may enhance pain-related distress regulation rather than inhibit it. Future research should explore ideal training initiatives with researchers and vaccinating clinicians, as well as explore psychometric properties to validate the measure’s use when used during vaccinations.

Another important future direction relates to predictors of the distress-promoting parenting behaviors. For example, by supporting parental mental health (e.g., depression, anxiety, parenting stress, trauma), one may be able to reduce the number of distress-promoting responses a parent uses with his or her distressed infant.

### Limitations

Past research by our lab^29,30^ has shown that there are rare cases of infants who do not respond with moderate to high levels of pain-related distress immediately post vaccine injection. In these cases, the presence of these eight distress-promoting behaviors becomes ambiguous post vaccine injection because it is unclear whether the infant is not signaling pain after the needle because of no pain or because he or she has learned that expressing distress to his or her parent does not elicit help (one hallmark of insecure attachments). It is critical that the set of behaviors only be coded in the presence of moderate-high infant distress. In addition, there was no experimental manipulation; thus, causation should not be inferred from the significant correlations in this work. Finally, because this was a low-risk sample, generalizability to higher risk samples must be established.

## Conclusion

In conclusion, this new measure appears to be a valid way to measure distress-promoting parent behaviors in the infant vaccination context. Measuring distress-promoting behaviors appears to be a novel and fruitful way to explore the relationships between caregiver behavior and infant pain. Not only does the measure’s feasibility allow for research use by a wider range of disciplines, but the potential for incorporation into primary care will allow for better parent coaching and support during painful procedures.
